# Effect of tumor shape, size, and tissue transport properties on drug delivery to solid tumors

**DOI:** 10.1186/1754-1611-8-12

**Published:** 2014-06-12

**Authors:** Mostafa Sefidgar, M Soltani, Kaamran Raahemifar, Hossein Bazmara, Seyed Mojtaba Mousavi Nayinian, Majid Bazargan

**Affiliations:** 1 Department of Mechanical Engineering, K. N. T. University of Technology, Tehran, Iran; 2 Division of Nuclear Medicine, Department of Radiology and Radiological Science, School of Medicine, Johns Hopkins University, Baltimore, MD, USA; 3 Electrical & Computer Department of Ryerson University, Toronto, Ontario

**Keywords:** Drug concentration, Solid tumor, Tumor shape and size, Tissue transport properties

## Abstract

**Background:**

The computational methods provide condition for investigation related to the process of drug delivery, such as convection and diffusion of drug in extracellular matrices, drug extravasation from microvessels or to lymphatic vessels. The information of this process clarifies the mechanisms of drug delivery from the injection site to absorption by a solid tumor. In this study, an advanced numerical method is used to solve fluid flow and solute transport equations simultaneously to investigate the effect of tumor shape and size on drug delivery to solid tumor.

**Methods:**

The advanced mathematical model used in our previous work is further developed by adding solute transport equation to the governing equations. After applying appropriate boundary and initial conditions on tumor and surrounding tissue geometry, the element-based finite volume method is used for solving governing equations of drug delivery in solid tumor. Also, the effects of size and shape of tumor and some of tissue transport parameters such as effective pressure and hydraulic conductivity on interstitial fluid flow and drug delivery are investigated.

**Results:**

Sensitivity analysis shows that drug delivery in prolate shape is significantly better than other tumor shapes. Considering size effect, increasing tumor size decreases drug concentration in interstitial fluid. This study shows that dependency of drug concentration in interstitial fluid to osmotic and intravascular pressure is negligible.

**Conclusions:**

This study shows that among diffusion and convection mechanisms of drug transport, diffusion is dominant in most different tumor shapes and sizes. In tumors in which the convection has considerable effect, the drug concentration is larger than that of other tumors at the same time post injection.

## Background

Cancer, the main cause of death in developed countries, is the second leading cause of death in developing countries [1]. Solid tumors account for 85% of human cancers [2]. Chemotherapy is one of the ways widely used for cancer treatment. Based on the findings from clinical applications, most cancer treatments with drugs fail to eliminate solid tumors completely [3]. The computational method can investigate why systemic administration cannot distribute drug uniformly in tumors. The drug exchange between microvessels and extracellular matrices, drug removal by lymphatic system, drug diffusion and convective transport in extracellular matrices should be included by mathematical simulation. Computational fluid dynamics (CFD) can model the whole drug delivery process and clarify the mechanisms of drug delivery from the injection site to absorption by a solid tumor.

Baxter and Jain, based on the theoretical framework in their 1D mathematical method, found the effective factors on drug delivery such as microvessel permeability, interstitial fluid pressure (IFP), and interstitial fluid velocity (IFV) [4-7].

Improving the 1D model of Baxter et al. [5,6,8] and Saltzman et al. [9], Wang et al. [10-12] developed a simulation framework of drug delivery to tumors by considering the complex 3D geometry. Wang and Li [10] used modified MRI images for tumor geometry. They considered interstitial fluid flow with blood and lymphatic drainage in their model. Wang et al. [11] studied the effect of elevated interstitial pressure, convective flux, and blood drainage on the delivery of specified solute to brain tumors.

The study of tissue transport property effect on drug delivery is considered in recent studies. Zhao et al. [13] used a 3D computational model to predict the distribution of IFV, IFP, and solute transport through a tumor. Arifin et al. [14] studied the sensitivity of drug distribution to physiochemical properties in realistic models of brain tumors. A specific tumor captured by MRI is used by Pishko et al. [15] for modeling drug distribution in tissue with spatially-varying porosity and vascular permeability. The sensitivity of solute distribution to tumor shape and size is not considered in above mentioned works.

In our previous work [2], tumor shape and size effect on drug delivery is investigated by modeling interstitial fluid flow and assuming that drug particles flow with the interstitial fluid. In the present work, by adding the solute transport equation to the previous developed model in our group [16-20], new governing equations are investigated to find drug concentration in interstitial fluid (DCIF). Solving fluid flow and solute transport equations simultaneously, the effects of tumor shape, size, and tissue transport properties on drug delivery to solid tumor are also investigated.

Spherical and non-spherical tumors and their surrounding normal tissue are modeled with assumption of rigid porous media. The vasculature as a source term and lymphatic vessel as a sink term vary spatially. In the following parts of this paper, the sensitivity analysis provides a better understanding of the effects of tissue transport parameters on drug delivery.

## Results

Simulation of interstitial fluid flow for baseline values (Table 1) predicts that IFP has its greatest value in the tumor center. IFP is non-dimensionalized by effective pressure. The effective pressure, *P*_*eff*_, is a parameter defined by intravascular pressure, plasma osmotic pressure, and interstitial osmotic pressure by Equation (1). The non-dimensionalized pressure is defined by Equation (2)

**Table 1:** Interstitial transport properties used in numerical simulations

**Parameter**	**Baseline value**	**Reference**
L_P_[cm/mmHg s]		
Normal	0.36 × 10^−7^	[16]
Tumor	2.80 × 10^−7^	
K[cm^2^/mmHg s]		
Normal	8.53 × 10^−9^	[16]
Tumor	4.13 × 10^−8^	
S/V[cm^−1^]		
Normal	70	[16]
Tumor	200	
P_B_[mmHg]		
Normal	15.6	[16]
Tumor	15.6	
π_B_[mmHg]		
Normal	20	[16]
Tumor	20	
π_i_[mmHg]		
Normal	10	[16]
Tumor	15	
σ		
Normal	0.91	[16]
Tumor	0.82	
P_L_		
Normal	0	[15]
L_PL_ S_L_/V [1/mmHg s]		
Normal	1.33 × 10^−5^	[15]

(1) $$P_{\mathrm{eff}}=P_{B}^{t}-\sigma_{s}^{t}\left(\pi_{B}^{t}-\pi_{i}^{t}\right)$$

(2) $$P_{n}=\frac{P_{i}}{P_{\mathrm{eff}}}=\frac{P_{i}}{P_{b}^{t}-\sigma_{s}^{t}\left(\pi_{b}^{t}-\pi_{i}^{t}\right)}$$

where t stands for tumor tissue. Parameters used in Equation (1) and (2) are introduced in the method section.

**Figure 1:**
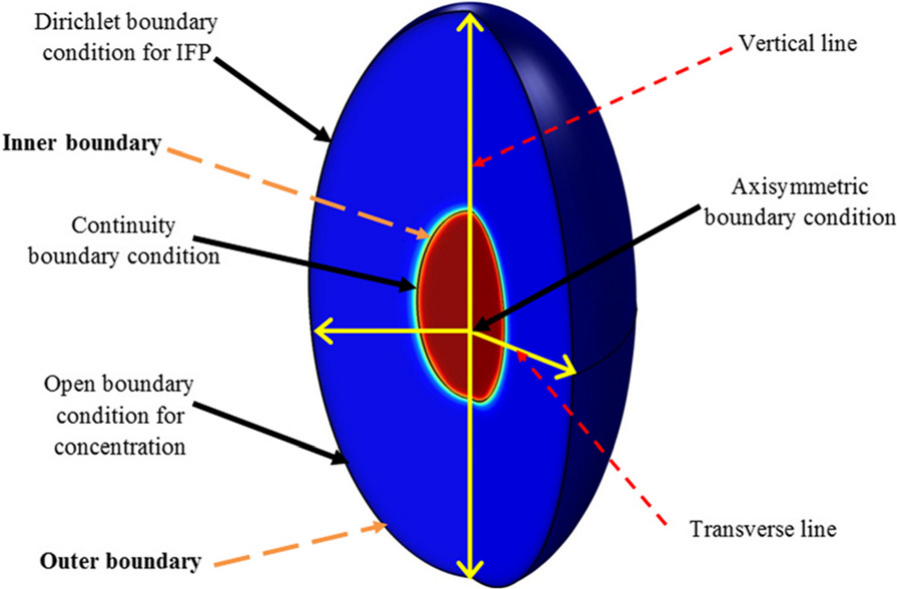
**Schematic of considered geometry and boundary conditions.** The transverse and vertical lines are used to show results along them.

**Figure 2:**
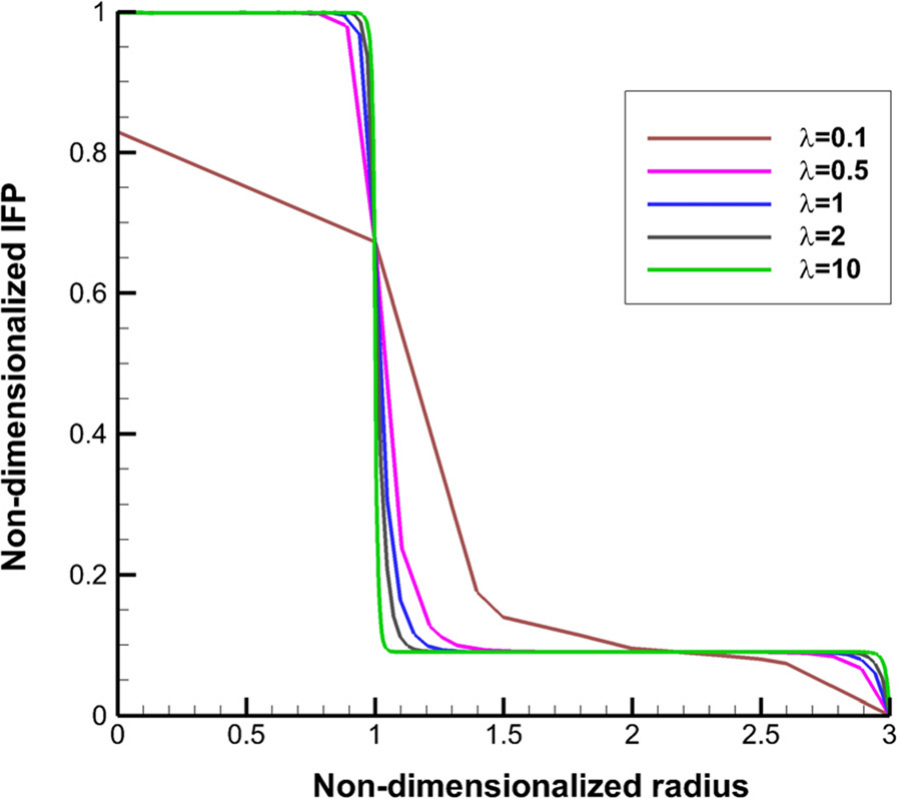
**Interstitial fluid pressure profile along transverse line.** The 1 in “x” axis shows the boundary of tumor (inner boundary).

**Figure 3:**
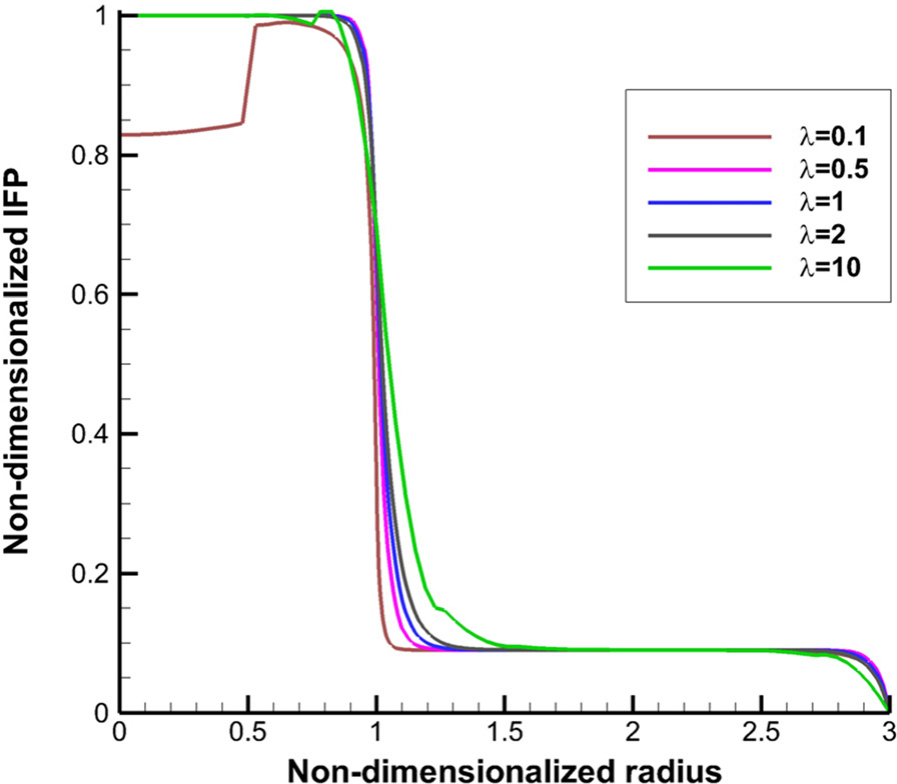
**Interstitial fluid pressure profile along vertical line.** The 1 in “x” axis shows the boundary of tumor (inner boundary).

Non-dimensionalized IFP along transverse and vertical lines (shown in Figure 1), are illustrated in Figures 2 and 3. The maximum value of IFP occurs in the tumor center (Figure 2). This value is equal to *P*_*eff*_, 1.53 kPa, except for the case of *λ* = 0.1 (Prolate shape). IFP has uniform distribution at tumor region and in the inner boundary (for more detail see Figure 1 and boundary condition section) IFP falls down sharply to around 150 Pa as shown in Figure 2 for all shapes except for prolate one (*λ* = 0.1). In normal tissue, pressure has uniform distribution close to outer boundary (for more detail, see Figure 1 and boundary condition section) then it decreases smoothly to peripheral pressure. For prolate shape as shown in Figure 2, IFP reduces smoothly from tumor center to the outer boundary, and IFP has a non-zero gradient in whole domain. Figure 2 shows the IFP along vertical line. Results are very similar to IFP results in Figure 2. Only for a prolate shape IFP has different pattern from what is observed in Figure 2. Maximum value of IFP in the prolate shape does not occur in the tumor center and takes place somewhere between the tumor center and tumor periphery.

**Figure 4:**
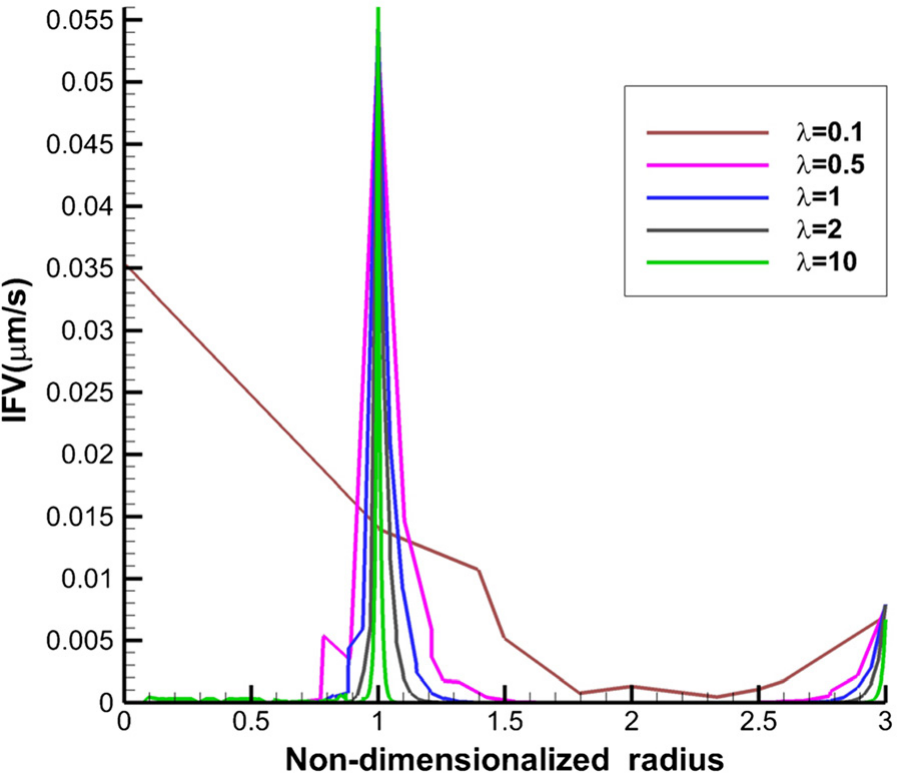
**Interstitial fluid velocity profile along transverse line.** The 1 in “x” axis shows the boundary of tumor (inner boundary).

**Figure 5:**
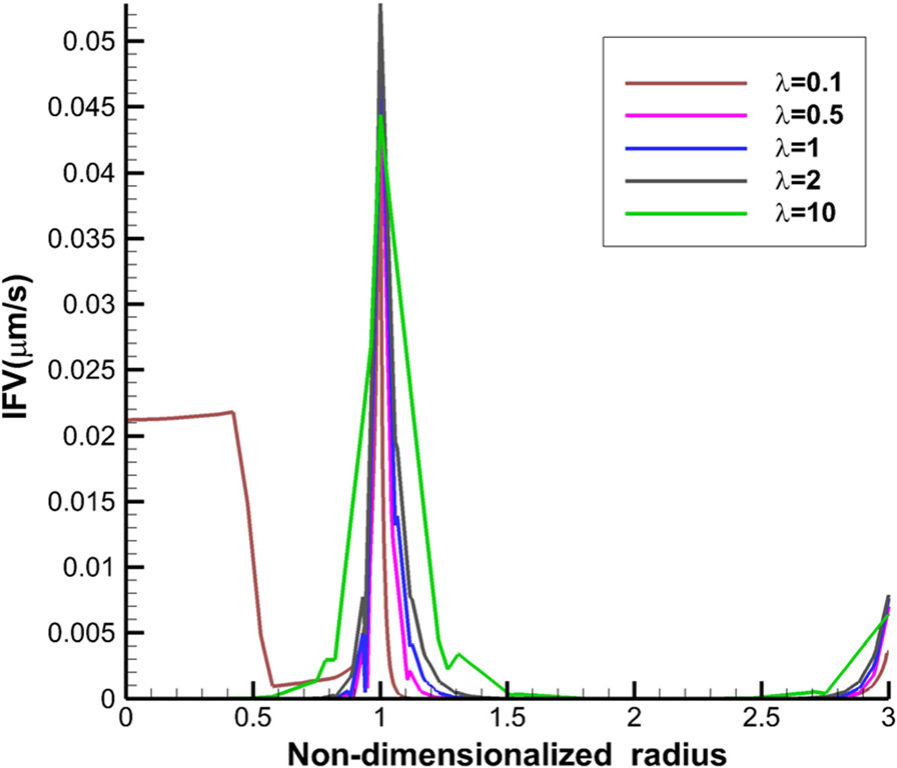
**Interstitial fluid velocity profile along vertical line.** The 1 in “x” axis shows the boundary of tumor (inner boundary).

IFV distribution along the vertical and transverse lines is presented in Figures 4 and 5. Maximum value of IFV occurs close to the inner boundary. Also, in normal tissue, IFV reaches zero far from the inner boundary. However, for the prolate shape, velocity is not zero, especially along transvers line (Figure 4).

**Figure 6:**
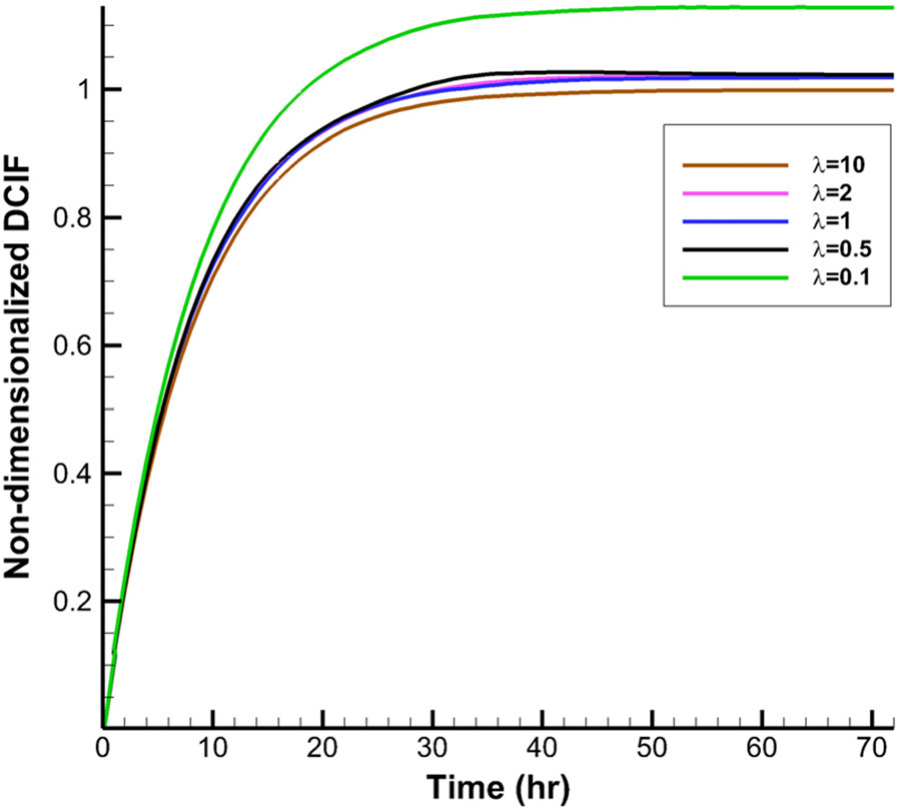
Average non-dimensionalized DCIF in tumor region in during time for continuous injection.

**Figure 7:**
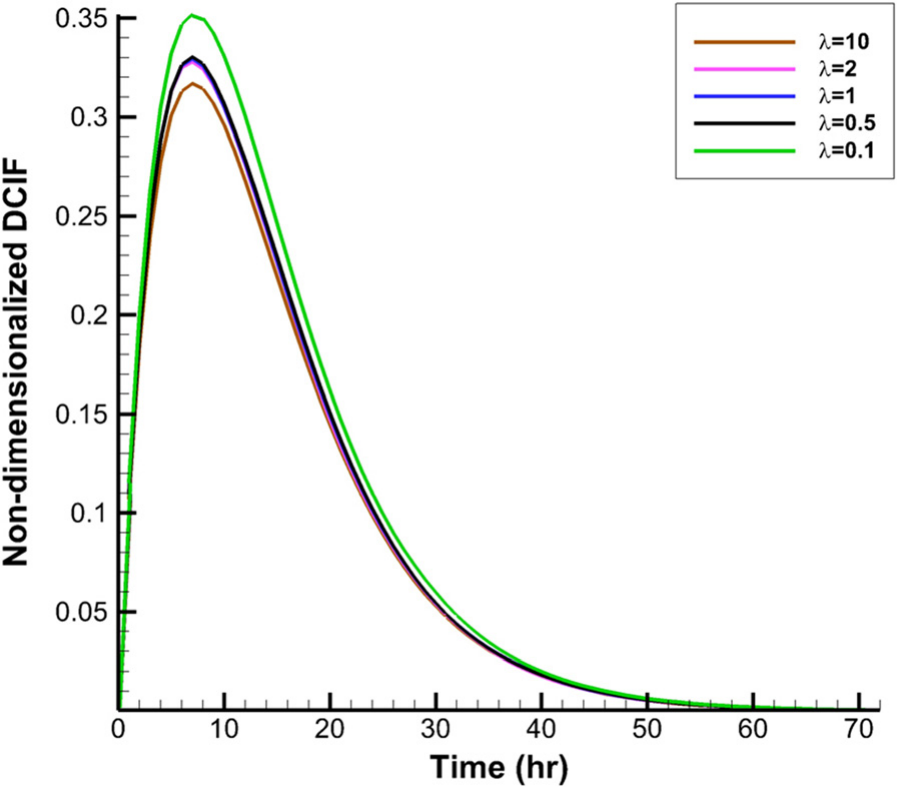
Average non-dimensionalized DCIF in tumor region in during time for bolus injection.

**Figure 8:**
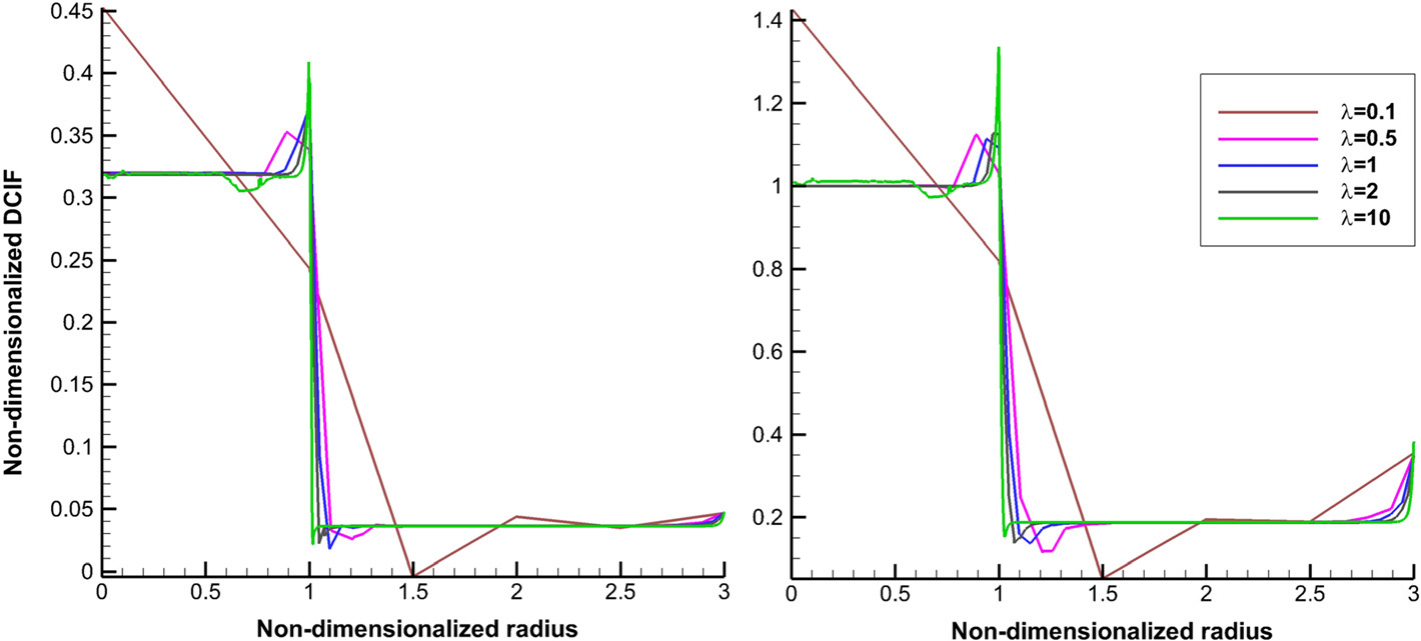
**Non-dimensionalized DCIF profile along transverse line for normal.** Left for bolus injection and right for continuous injection. The 1 in “x” axis shows the boundary of tumor (inner boundary).

**Figure 9:**
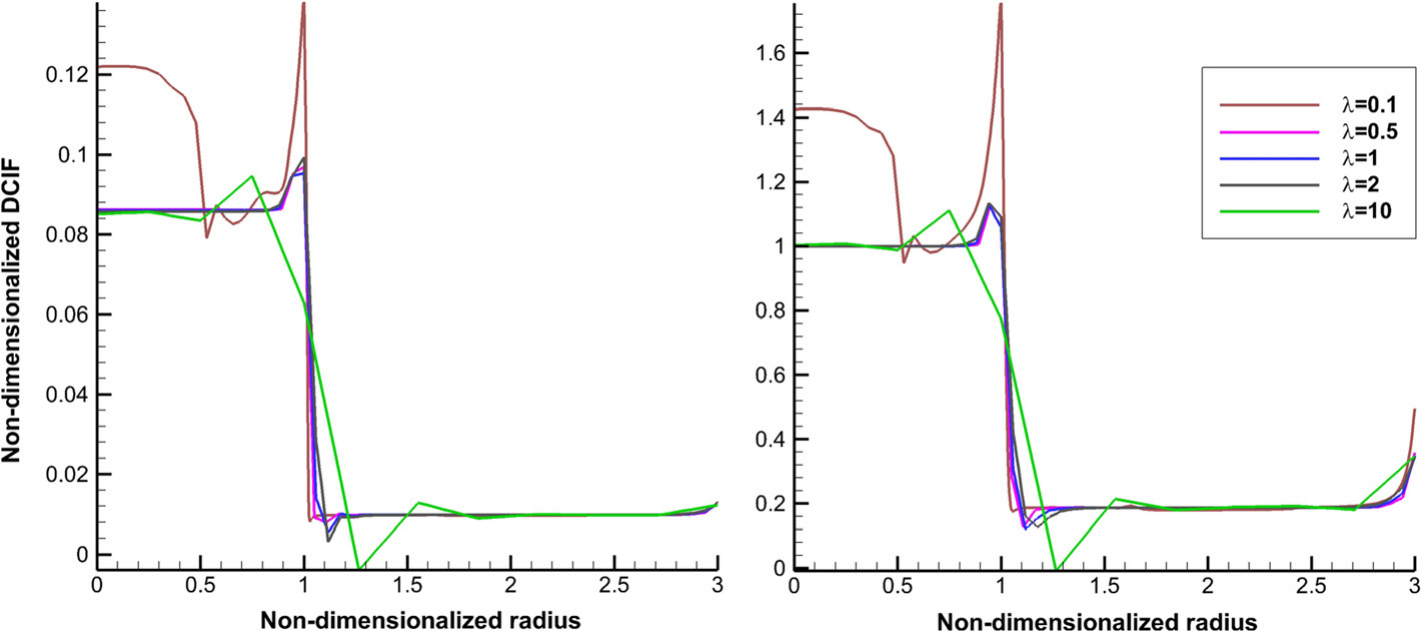
**Non-dimensionalized DCIF profile along vertical line.** Left for bolus injection and right for continuous injection. The 1 in “x” axis shows the boundary of tumor (inner boundary).

DCIF is simulated in two cases of injection. In the first case, the continuous injection which leads to constant plasma concentration (*C*_*p*_ = constant) is considered and in the second case, the bolus injection in which the plasma concentration decreases with time exponentially ($C_{p}=C_{p}^{0}e^{-\ln\left(0.5\right)t/\tau }$) is studied, in which *τ* is the drug half-life in plasma (Table 2). DCIF are non-dimensionalized by *C*_*P*_ for continuous injection and $C_{P}^{0}$ for bolus injection, respectively. Average of non-dimensionalized DCIF for two injection cases in different times is shown in Figures 6 and 7. DCIF of prolate shape (*λ* = 0.1) has the maximum value and DCIF of oblate shape (*λ* = 10) has the minimum value. Other considered shapes show the similar transient behavior.Non-dimensionalized DCIF along two lines (transverse and vertical) is illustrated in Figures 8 and 9. The bolus injection results are presented in 8 hr post injection in which the concentration is maximum based on Figure 7 and for continuous injection is presented at final time of simulation, in 72 hr post injection. The profiles of two types of injections are similar in spite of different values of DCIF. As observed in IFV and IFP profiles, the prolate shape is different from the other shapes in DCIF distribution. A bump is observed in DCIF curves at the inner boundary of all tumor shapes. The normal tissue has uniform distribution of DCIF except near the boundaries (inner and outer). DCIF distribution in normal tissue is the same for all tumor shapes.

**Table 2:** Parameters of solute transport model used in numerical simulations

**Parameter**	**Baseline value**	**Reference**
σ_f_		
Normal	0.9	[8]
Tumor	0.9	
*D*_*eff*_ [m^2^/s]		
Normal	0.16× 10^−12^	[8]
Tumor	2.0× 10^−12^	
P[m/s]		
Normal	2.2× 10^-10*^	[8]
Tumor	17.3× 10^-10*^	
τ [hr]^**^		
Plasma	6.1	[8]

*- the microvessel permeability coefficient is 10% of effective permeability introduced in [8].

**- the drug half-life in plasma introduced in results section.

**Figure 10:**
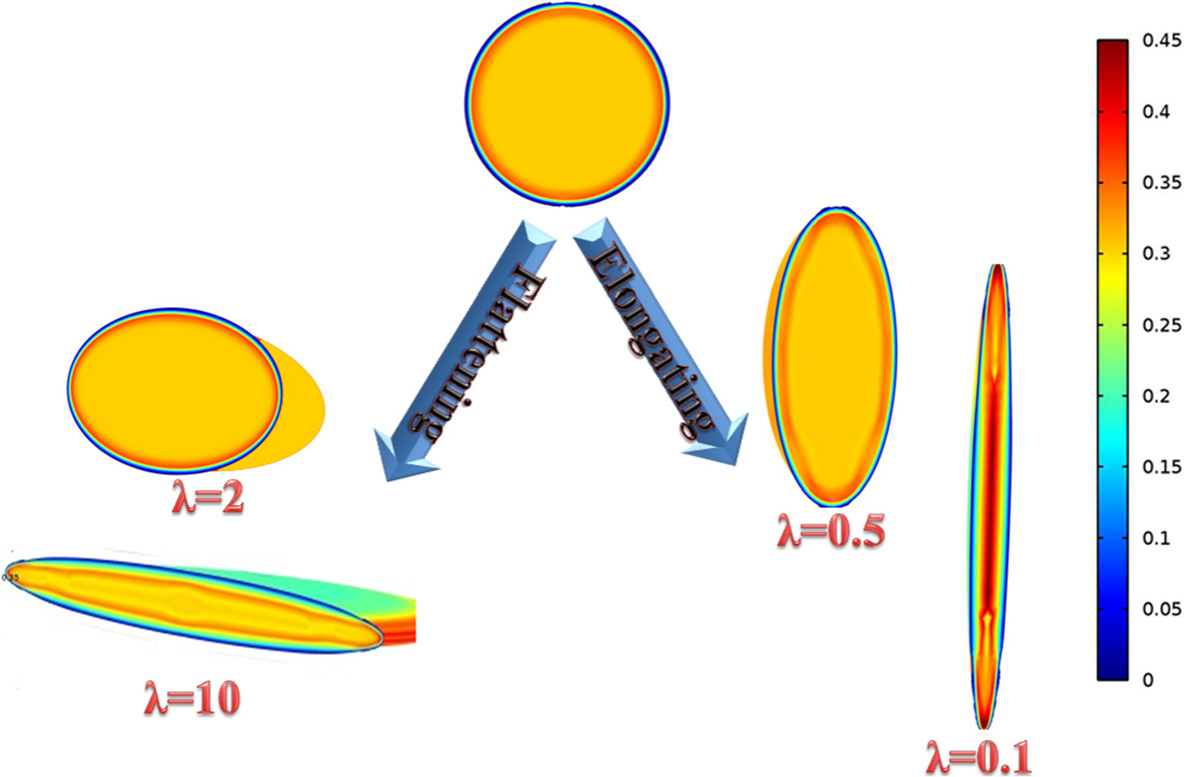
2D contour of DCIF in tumor region for bolus injection in 8 hr post injection for all tumor shapes.

**Figure 11:**
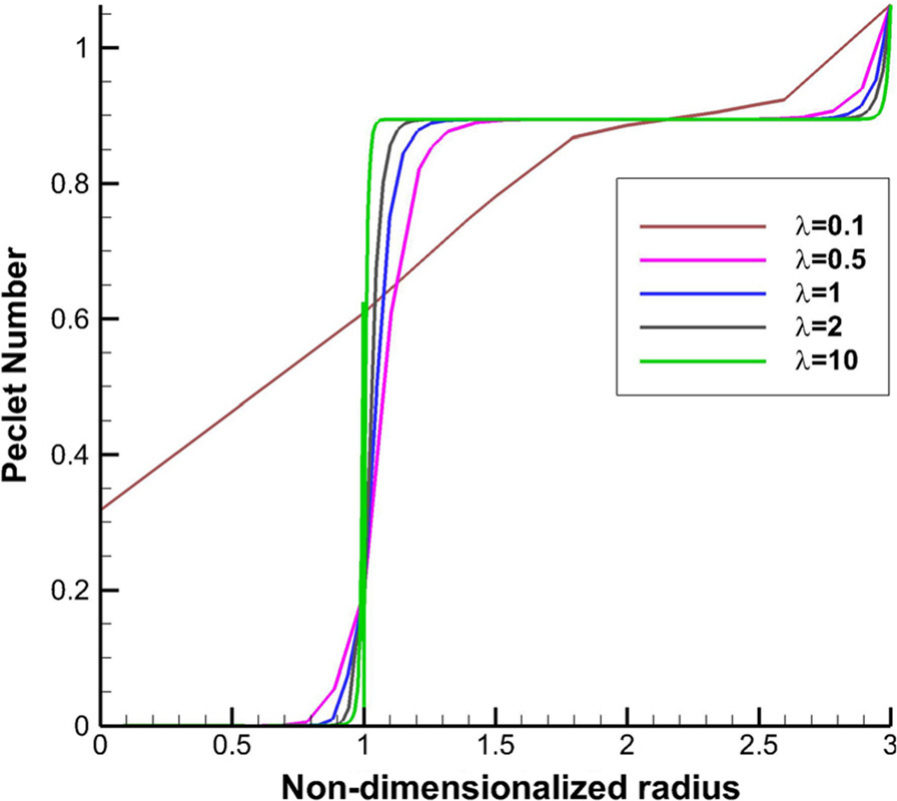
Peclet number (ratio of convection to diffusion across the microvessel wall) along transverse line.

**Figure 12:**
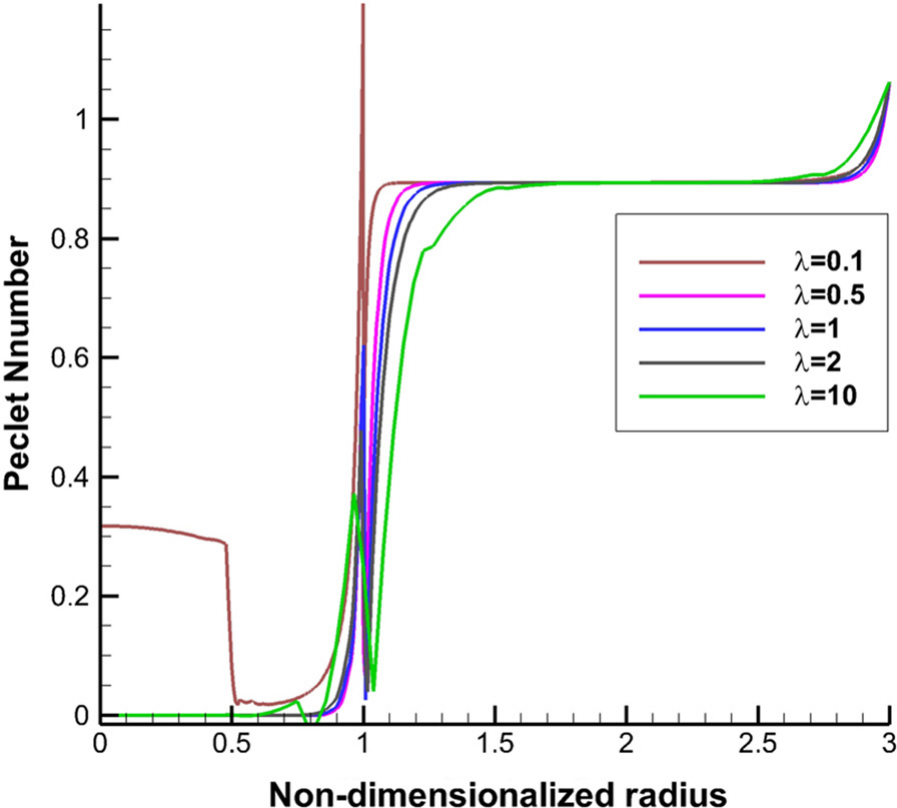
Peclet number (ratio of convection to diffusion across the microvessel wall) along vertical line.

2D contours of DCIF in tumor region for bolus injection in 8 hr post injection are shown in Figure 10 for all tumor shapes. Results show that DCIF for tumors with *λ* = 0.1 and *λ* = 10 has different distributions. Generally, in the inner boundary, DCIF has its maximum value. In Figures 11 and 12, Peclet number distribution along two lines for continuous injection is shown. Peclet number demonstrates the ratio of convection to diffusion across the microvessel wall. Results show that in the tumor region Peclet number is zero except for prolate shape. Peclet number for the prolate shape especially along short radius (transverse line) is greater than zero.

Some of tissue transport parameters mentioned (effective pressure, hydraulic conductivity, and tumor size) in Table 1 are investigated for sensitivity analysis. The values of these parameters are selected near the ranges reported in the literature [2,15,21].

**Figure 13:**
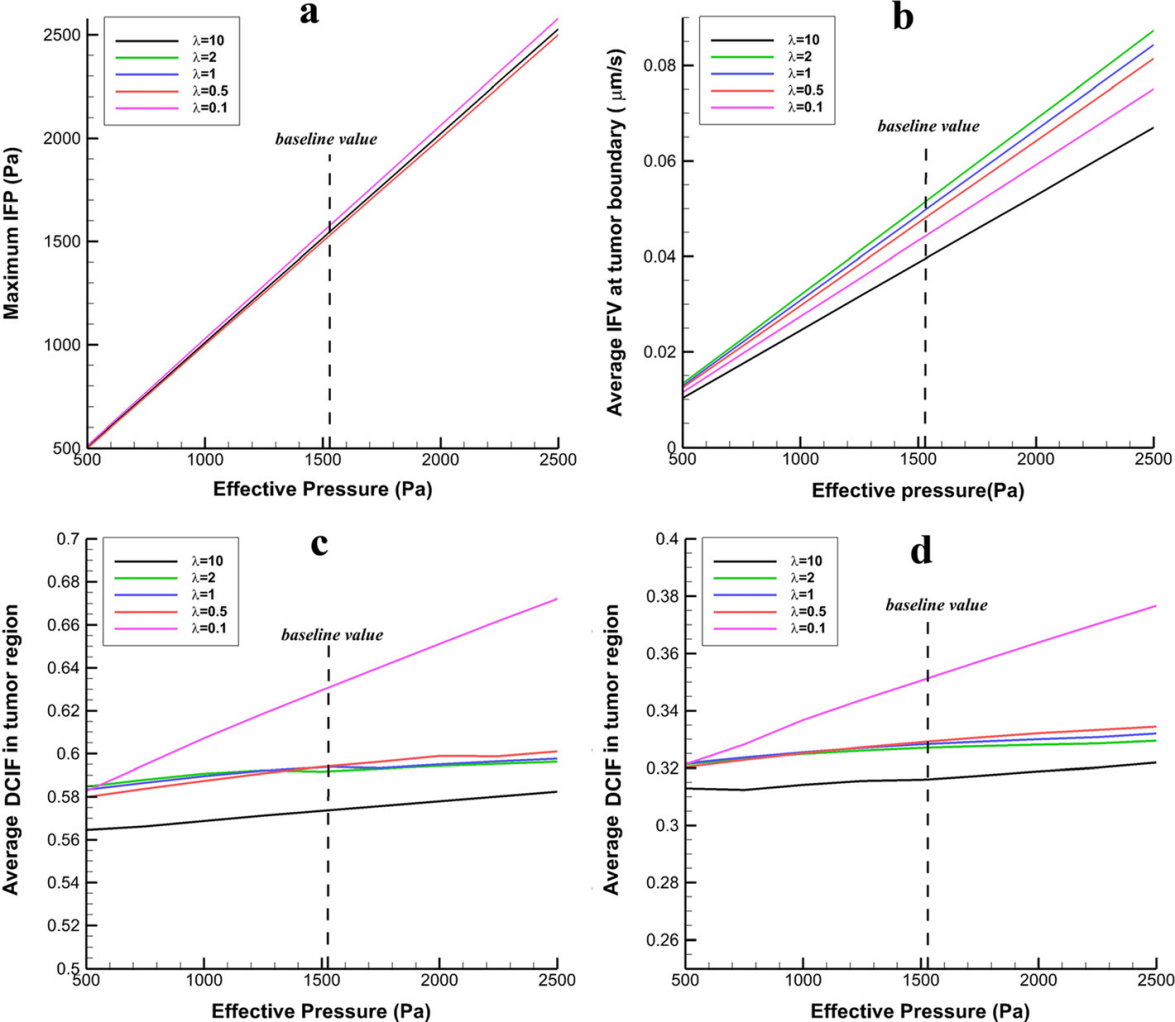
**The effect of different values of effective pressure on IFP, IFV and DCIF. a)** The variation of maximum IFP in tumor region. **b)** The variation of averaged IFV at tumor boundary. **c)** Average of DCIF in tumor region for continuous injection. **d)** Average of DCIF in tumor region for bolus injection.

Figure 13 shows the influence of changing effective pressure, *P*_*eff*_, on numerical results of tumor shapes with the same volume. Maximum value of IFP in tumor region (Figure 13a) linearly increases when *P*_*eff*_ increases. Only in the prolate shape the pressure increases more than other tumor shapes. Average of IFV on inner boundary also has the same pattern and linearly changes with *P*_*eff*_ (Figure 13b). The changes in average of DCIF in tumor region for two cases of injection are shown in Figure 13c and d. For these two cases of injection, the prolate shape is more sensitive to *P*_*eff*_ changes than other tumor shapes and DCIF changes around 20% when the *P*_*eff*_ changes from 500 Pa to 2500 Pa. The other shapes do not show significant variations in DCIF for these ranges of *P*_*eff*_.

Different tumor sizes are studied with changes in their volume. One of important metric of disease development and response to tumor therapy with drug is volume of tumors [22-25]. To quantify response to therapeutic regimens and also assess disease progression, tumor volume is used as a metric in many studies, such as Char et al. [26], Jensen et al. [27] and Gass et al. [28].

**Figure 14:**
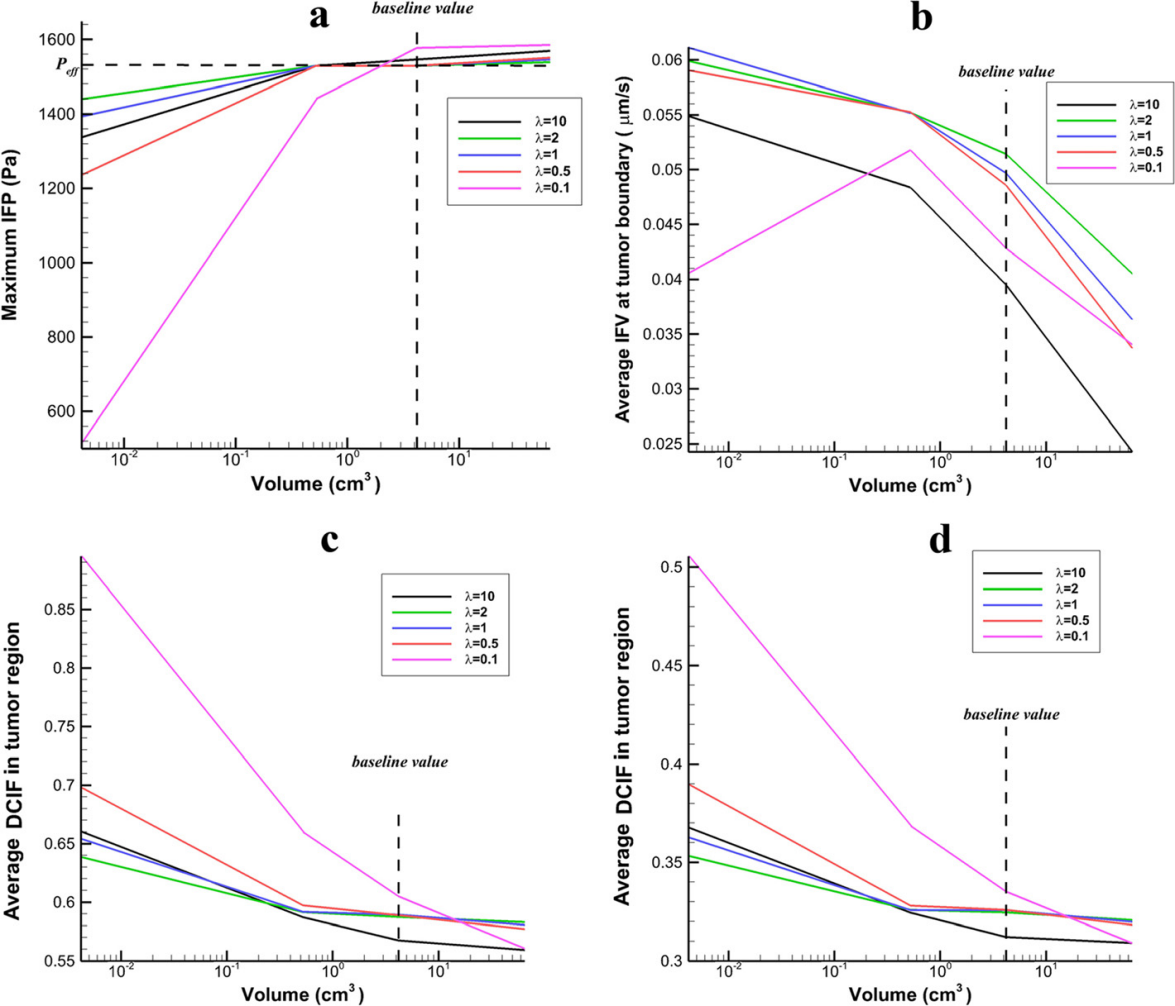
**The effect of different values of tumor size on IFP, IFV and DCIF. a)** The variation of maximum IFP in tumor region. **b)** The variation of averaged IFV at tumor boundary. **c)** Average of DCIF in tumor region for continuous injection. **d)** Average of DCIF in tumor region for bolus injection.

**Figure 15:**
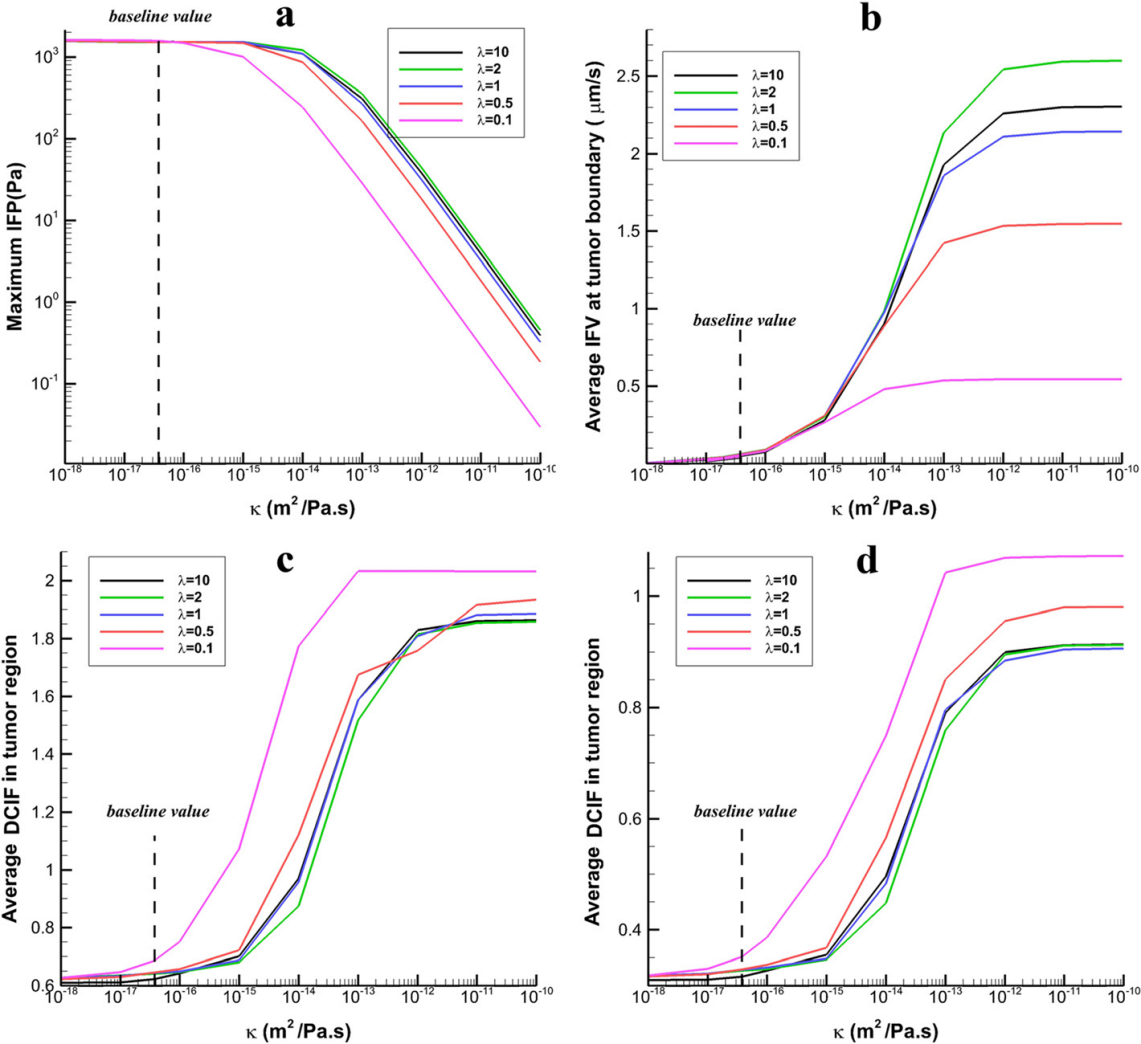
**The effect of different values of hydraulic conductivity on IFP, IFV and DCIF. a)** The variation of maximum IFP in tumor region. **b)** The variation of averaged IFV at tumor boundary. **c)** Average of DCIF in tumor region for continuous injection. **d)** Average of DCIF in tumor region for bolus injection.

As shown in Figure 14a, IFP has less value than *P*_*eff*_ when the tumor volume is smaller than 1 cm^3^. When the tumor volume is in the order of 1 cm^3^, IFP reaches *P*_*eff*_ and by increasing the tumor radius, IFP remains constant. Average of IFV on the inner boundary generally decreases with increasing tumor size, Figure 14b. Only in prolate shape in small radii, IFV increases with the tumor size. As shown in Figure 14c and d, the mean values of DCIF have the greatest value in the smallest tumor. Also, the prolate shape has the highest value of DCIF in all studied tumors.Figure 15 shows the sensitivity of IFF parameters and DCIF to hydraulic conductivity changes. Results show that in all tumor shapes, if hydraulic conductivity increases, the maximum value of IFP remains constant and then decreases sharply. Average of IFV increases by increasing hydraulic conductivity. DCIF increases by increasing hydraulic conductivity and then reaches a constant value in spite of increasing hydraulic conductivity.

## Discussion

This study presents DCIF, IFP, and IFV in solid tumors surrounded by normal tissue in two types of injection; bolus and continuous one. The model used in this study investigates the effect of two characteristics of tumors on concentration distribution; tumor shape and size.

Results of high IFP in tumors are discussed in our previous studies [2,16,17] and in the experimental results of Arifin et al. [29] and Huber et al. [30]. Maximum value of IFP in spherical tumors (1 cm radius) for baseline values in Table 1 is 1529.5 Pa which is close to the studies of Jain et al. [31], Chauhan et al. [32], and Arfin et al. [33]. The current results are verified by experimental data of IFP measured by Nielsen et al. [34]. In their work, the wick-in-needle technique is used to measure IFP in two types of tumors with the same volume as tumors in the current study. They found IFP in the range of 1400 Pa to 1600 Pa.

IFV on tumor boundary in spherical tumors with baseline values is around 0.05 *μm*/*s* which is at the same order of the prediction of Jain et al. [31] and experimental observation of Hompland et al. [35]. Also, the profile of drug concentration for simulation with baseline values for spherical tumor in different times (Figures 6 and 7) agrees well with Baxter and Jain’s predictions [8]. Results show that DCIF for prolate shape (*λ* = 0.1) always has the greatest value. Results of transient DCIF (Figures 6 and 7) show that drug delivery is much easier in the prolate tumors. Also, the oblate shape (*λ* = 10) has the most resistance to drug delivery. Following of this section, the reason of these phenomena is investigated.

The uniform values of IFP for all tumor shapes except prolate one (Figures 2 and 3) is equal to *P*_*eff*_. Non-uniform IFP in prolate shape results in the maximum value of DCIF among other tumor shapes with the same volume. Equation (17) is able to legitimize this behavior of tumor shapes. The source term (the last term in the right hand side of Equation (17)) includes diffusion and convection terms. The convection term depends on the differences between IFP and *P*_*eff*_ based on starling’s law. This pressure difference is close to zero (Figures 2 and 3) for all tumor shapes except for the prolate one and therefore the convection term only for prolate shape has non-zero value. Because of the non-zero values of Peclet number for prolate shape in tumor region (Figures 11 and 12), the concentration for this shape is affected not only by diffusion rate from vessels but also by convection rate from vessels. The convection rate leads to higher level of DCIF in prolate shape than that of other tumor shapes (Figures 6, 7, 8, 9 and 10). The non-zero values of Peclet number for prolate shape are seen in an image based work of Zhao et al. as well [13]. In normal tissue, Peclet number shows that drug delivery from microvessel to tissue is done by both mechanisms of transfer, convection and diffusion.

The other effect of uniform pressure is zero pressure gradients in all tumor shapes except for prolate one. Because of zero IFP gradient and based on Darcy’s equation, IFV is close to zero in tumor tissue except for prolate shape. The close to zero value of IFV is predicted in a few numerical studies such as Welter and Rieger [36]; Roy and Riahi [37]; and experimental results of Hompland et al. [35]. Zero IFV results in a negligible convection transport (the second term of the right hand side of Equation (17)) and consequently the convection transport does not affect drug distribution; and the diffusion transport (the first term of the right hand side of Equation (17)) is the only reason of drug transport in all tumor shapes except for prolate one. Therefore, non-zero IFV in prolate shape, also seen in Zhao et al. [13], is another reason of higher DCIF values in this tumor shape with respect to other shapes.The sharp pressure gradient (Figures 2 and 3) and highest value of IFV (Figures 4 and 5) in the inner boundary for all tumor shapes increases drug transport and make a bump at this boundary for DCIF.

The sensitivity analysis of effective pressure shows that the effective pressure does not have too much effect on DCIF. In all tumor shapes, DCIF for a wide range of effective pressure changes smoothly; however, in prolate shape, this change is sharper and increases by effective pressure (Figure 13c and d). As mentioned, in the tumor region for all tumor shapes except for prolate one the convection rate, which depends on *P*_*eff*_, has a negligible role on drug distribution; therefore, *P*_*eff*_ cannot have significant effect on drug concentration. However, in prolate shape, increasing *P*_*eff*_ increases pressure difference between IFP and *P*_*eff*_ and consequently the convection rate from vessels in the tumor region; therefore, DCIF in prolate shape is sensitive to *P*_*eff*_ changes.

The tumor volume shows more effects on IFP, IFV, and DCIF than other investigated parameters such as effective pressure and hydraulic conductivity. The increasing tumor volume increases significantly IFP (Figure 14a). The dependency of IFP to tumor volume is observed in experimental study of Gutmann et al. [38], Hompland et al. [39], and Leguerney et al. [40], as well. When the tumor volume is in the order of 1 cm^3^, the sensitivity of IFP to tumor size decreases. The independency of IFP to tumor volume in in large tumors is observed in the study of Leguerney et al. [40], as well. In their work, IFP changes very slowly with tumor volume. Since the high IFP is introduced as the main barrier of drug delivery to tumors, IFP increasing with tumor volume leads to DCIF decrease in these tumors. This reduction of DCIF with tumor size is observed in Au et al. [41], as well. Since IFP reaches around the effective pressure with increasing tumor volume, the convection rate is vanished and the diffusion rate reaches a constant value, and consequently the sensitivity of DCIF to tumor size reduces. Lower IFP in the small tumor sizes leads to increase the convection rate of source term in Equation (17). Therefore, it is expected to have a better drug distribution in small tumors.

Results show that IFV, IFP, and DCIF are sensitive to tissue hydraulic conductivity changes. The hydraulic conductivity is appeared only in Darcy’s law. This parameter has a direct effect on IFF and indirect effect on DCIF. Theoretical analysis shows that the $\frac{P_{i}}{P_{\mathrm{eff}}}$ in tumor region is proportional to $1-\frac{1}{\sqrt{\kappa }}/\sinh\left(\frac{1}{\sqrt{\kappa }}\right)$[8] (*κ* is hydraulic conductivity, see material section). In low values of *κ*, the $\frac{1}{\sqrt{\kappa }}/\sinh\left(\frac{1}{\sqrt{\kappa }}\right)$ is negligible and *P*_*i*_ is close to *P*_*eff*_ (Figure 15a). Increasing *κ*, increases the $\frac{1}{\sqrt{\kappa }}/\sinh\left(\frac{1}{\sqrt{\kappa }}\right)$ and leads to sharp decrease in IFP. This dependency is also observed by McCarty and Johnson [42]. For high values of *κ*, IFP is very low and negligible in comparison to *P*_*eff*_. IFP reduction from effective pressure increases IFV around 5 times (Figure 15b). The hydraulic conductivity affects DCIF by convection rate from vessels (as mentioned this value depends on difference between IFP and *P*_*eff*_). In low values of *κ*, since IFP is equal to *P*_*eff*_, the effect of convection rate is not significant and DCIF remains constant. Increasing *κ* increases the convention rate and consequently DCIF.

When hydraulic conductivity increases two to three orders of magnitude, the mean values of DCIF are two times greater than the average of DCIF for baseline values in Table 1. However, after a specific value of hydraulic conductivity, DCIF changes smoothly and reaches a constant value because IFP is very low and convection rate only depends on *P*_*eff*_.

## Conclusions

A numerical approach which couples the mathematical model of the lymphatic system and the interstitial flow with the mathematical model of solute transport demonstrates that DCIF is affected by two transport mechanisms, convection and diffusion.

Drug convection and drug transport from microvessel to tumor are blocked by high interstitial pressure (IFP) which is uniformly distributed in most part of the tumor. The large pressure gradient results in an outward convective flow that washes out the drug extravasated from microvessels at the tumor periphery. This study shows that when there is IFP gradient in the tumor region instead of uniform IFP distribution which occurs in some tumor shapes, DCIF is greater than that of the uniform one.

As the effects of osmotic and intravascular pressure on convection rate are negligible in most of tumor shapes, the dependency of DCIF to these parameters is very low.

The hydraulic conductivity which is another considered parameter in sensitivity analysis has significant effect on drug distribution since it increases the convection rate from vessels.

## Method

### Mathematical model of interstitial fluid transport

This section introduces the mathematical model of interstitial fluid transport in macroscopic scale [2,16]. Since normal and tumor tissue have characteristics the same as porous media, fluid flow behavior is defined by coupling the fluid flow governing equations. The mass balance or continuity equation for steady state incompressible fluid in the porous media with source and sink of mass is [16]:

(3) $$\nabla .v_{i}=\phi_{B}-\phi_{L}$$

where

*v*_*i*_: The interstitial fluid velocity,

*ϕ*_*B*_: The source term, extravasation from microvessels, and

*ϕ*_*L*_: The drainage term, elimination by lymphatic system.

In biological tissues, the fluid source is evaluated through Starling's law as follows [16]:

(4) $$\phi_{B}=\frac{L_{P}S}{V}\left(P_{B}-P_{i}-\sigma_{s}\left(\pi_{b}-\pi_{i}\right)\right)$$

where

*P*_*i*_ : Interstitial fluid pressure,

*P*_*B*_ : Blood pressure in microvessel,

$\frac{S}{V}$ : The surface area per unit volume of tissue for transport in the interstitium,

*π*_*B*_: Microvessel oncotic pressure,

*π*_*i*_ : Interstitial oncotic pressure,

*L*_*p*_ : The hydraulic conductivity of the microvessel wall, and

*σ*_*s*_: Osmotic reflection coefficient.

and the lymphatic system is related to the pressure difference between the interstitium and the lymphatic vessels and is considered only for normal tissues [43]:

(5) $$\phi_{L}\left(r\right)=\left\{\begin{array}{ll} \frac{L_{\mathrm{PL}}S_{L}}{V}\left(P_{i}-P_{L}\right) & \mathrm{Normal}\;\mathrm{tissue}\\ 0 & \mathrm{Tumor}\;\mathrm{tissue} \end{array}\right)$$

where

*ϕ*_*L*_: The volumetric flow rate into the lymphatic,

$\frac{L_{\mathrm{PL}}S_{L}}{V}$: The lymphatic filtration coefficient, and

*P*_*L*_: The hydrostatic pressure of the lymphatic.

The momentum balance equation in its general form can be written as Equation (6) [44]:

(6) $$\begin{array}{ll} \;\rho \;\left(\frac{\partial v_{i}}{\partial t}+\left(v_{i}.\nabla \right)v_{i}\right)=\nabla \cdot [-P_{i}+\mu \left(\nabla v_{i}+{\left(\nabla v_{i}\right)}^{T}\right) & -\frac{2\mu }{3}\left(\nabla \cdot v_{i}\right)]\\ & -\left(\frac{\mu }{K}\right)v_{i}+F \end{array}$$

where

K: The permeability of the porous medium,

ρ : The density,

μ: The viscosity, and

F: The volume forces.

Since interstitial fluid is a Newtonian fluid and has low velocity through the tissues, Equation (6) in the steady state condition is simplified to Darcy’s law [16]:

(7) $$\nabla P_{i}=-\left(\frac{\mu }{K}\right)v_{i}$$

The K/μ is defined as hydraulic conductivity of the interstitium, *κ*:

(8) $$v_{i}=-\kappa \nabla P_{i}$$

Combination of momentum equation (Equation (8)) and the continuity equation (Equation (3)) results in

(9) $$-\nabla \cdot \left(\kappa \nabla P_{i}\right)=\phi_{B}-\phi_{L}$$

When *κ* is constant, the interstitial pressure can be expressed by

(10) $$-\kappa \;\nabla^{2}P_{i}=\phi_{B}-\phi_{L}$$

By substituting Equations (4) and (5) into Equation (10):

(11) $$-\kappa \;\nabla^{2}p=\left\{\begin{array}{ll} \frac{L_{P}S}{V}\left(P_{B}-P_{i}-\sigma_{s}\left(\pi_{b}-\pi_{i}\right)\right)-\frac{L_{\mathrm{PL}}S_{L}}{V}\left(P_{i}-P_{L}\right) & \mathrm{Normal}\;\mathrm{tissue}\\ \frac{L_{P}S}{V}\left(P_{B}-P_{i}-\sigma_{s}\left(\pi_{b}-\pi_{i}\right)\right) & \mathrm{Tumor}\;\mathrm{tissue} \end{array}\right)$$

### Macroscopic solute transport

Molecular transport in tumors is based on the conservation laws for mass and momentum. The interstitial transport of drug is governed by the convection diffusion equation; therefore, the general equation for the mass balance of solutes can be written as:

(12) $$\frac{\partial C}{\partial t}=\nabla \cdot \left(D_{\mathrm{eff}}\cdot \nabla C\right)-\nabla \cdot \left(v_{i}C\right)+\left(\Phi_{B}-\Phi_{L}\right)$$

where

*C* : The solute concentration based on tissue volume,

Φ_*B*_ : The rate of solute transport per unit volume from microvessel into the interstitial space,

Φ_*L*_ : The rate of solute transport per unit volume from the interstitial space into lymphatic vessels, and

*D*_*eff*_ : The effective diffusion tensor.

For an isotropic and uniform diffusion in tissues, equation (12) can be written as:

(13) $$\frac{\partial C}{\partial t}=D_{\mathrm{eff}}\nabla^{2}C-\nabla \cdot \left(v_{f}C\right)+\left(\Phi_{B}-\Phi_{L}\right)$$

The solute transport rate across the lymphatic vessels can be considered as [15]

(14) $$\Phi_{L}=\left\{\begin{array}{cc} \phi_{L}C & \mathrm{Normal}\;\mathrm{Tissue}\\ 0 & \mathrm{Tumor}\;\mathrm{Tissue} \end{array}\right)$$

The solute transport rate across the microvessel can be represented by Patlak equation [45]:

(15) $$\Phi_{B}=\phi_{B}\left(1-\sigma_{f}\right)C_{P}+\frac{\mathrm{PS}}{V}\left(C_{P}-C\right)\frac{\mathrm{Pe}}{e^{\mathrm{Pe}}-1}$$

where

(16) $$\mathrm{Pe}=\frac{\phi_{B}\left(1-\sigma_{f}\right)V}{\mathrm{PS}}$$

*ϕ*_*B*_: The fluid flow rate per unit volume of tissue across the microvessel wall,

*σ*_*f*_ : The filtration reflection coefficient,

*P* : The microvessel permeability coefficient,

*S/V* : The microvessel surface area per unit volume of tissue,

*C*_*p*_ : Solute concentration in the plasma.

By substituting Equations (15) and (14) in to Equation (13):

(17) $$\left\{\begin{array}{l} \begin{array}{l} \frac{\partial C}{\partial t}=D_{\mathrm{eff}}\nabla^{2}C-\nabla \cdot \left(v_{i}\mathrm{fC}\right)\\ \;+\left(\phi_{B}\left(1-\sigma_{f}\right)C_{P}+\frac{\mathrm{PS}}{V}\left(C_{P}-\frac{C}{K_{\mathrm{AV}}}\right)\frac{\mathrm{Pe}}{e^{\mathrm{Pe}}-1}-\phi_{L}C\right)\;\mathrm{Normal}\;\mathrm{region} \end{array}\\ \begin{array}{l} \frac{\partial C}{\partial t}=D_{\mathrm{eff}}\nabla^{2}C-\nabla \cdot \left(v_{i}\mathrm{fC}\right)\\ \;+\left(\phi_{B}\left(1-\sigma_{f}\right)C_{P}+\frac{\mathrm{PS}}{V}\left(C_{P}-\frac{C}{K_{\mathrm{AV}}}\right)\frac{\mathrm{Pe}}{e^{\mathrm{Pe}}-1}\right)\;\mathrm{Tumor}\;\mathrm{region} \end{array} \end{array}\right)$$

### Boundary conditions

A tumor surrounded by normal tissue is considered in this study. The tumor shape is considered ellipsoid. A 2D section of the geometry and boundaries are shown in Figure 1. The three boundaries are indicated for geometry: a) the center of tumor, b) the boundary between tumor and normal tissue, named inner boundary, c) the boundary at the outer edge of geometry, named outer boundary. The appropriate boundary conditions are implemented for Equations (11) and (17). The no flux boundary condition is applied at the center of the tumor; i.e. [16],

(18) $$\nabla P_{i}=0\;\mathrm{for}\;r=0$$

(19) $$D_{\mathrm{eff}}\nabla C+v_{i}C=0\;\mathrm{for}\;r=0$$

The continuity of pressure and velocity for Equation (11) and concentration and its flux for Equation (17) are considered as appropriate boundary conditions for inner boundary:

(20) $$\begin{array}{l} {\left(-\kappa_{t}\nabla P_{i}\right|}_{\Omega^{-}}={\left(-\kappa_{n}\nabla P_{i}\right|}_{\Omega^{+}}\\ {\left(P_{i}\right|}_{\Omega^{-}}={\left(P_{i}\right|}_{\Omega^{+}} \end{array}$$

(21) $$\begin{array}{l} {\left(\left(D_{\mathrm{eff}}^{t}\nabla C+v_{i}C\right)\right|}_{\Omega^{-}}={\left(\left(D_{\mathrm{eff}}^{n}\nabla C+v_{i}C\right)\right|}_{\Omega^{-}}\\ {\left(C\right|}_{\Omega^{-}}={\left(C\right|}_{\Omega^{+}} \end{array}$$

where *Ω*^−^ and *Ω*^+^ indicate the tumor and normal tissue at the inner boundary.

For outer boundary, that the interstitial pressure is constant; the Dirichlet type of boundary condition must be applied [16]:

(22) $$P_{i}=\text{for outer region}$$

And for concentration, the open boundary condition is used in the outer region [46]. The Open Boundary is used to set up mass transport across boundaries where both convective inflow and outflow can occur and defined by Equation (23):

(23) −n·∇C=0

where **n** is the normal vector.

### Geometry

**Figure 16:**
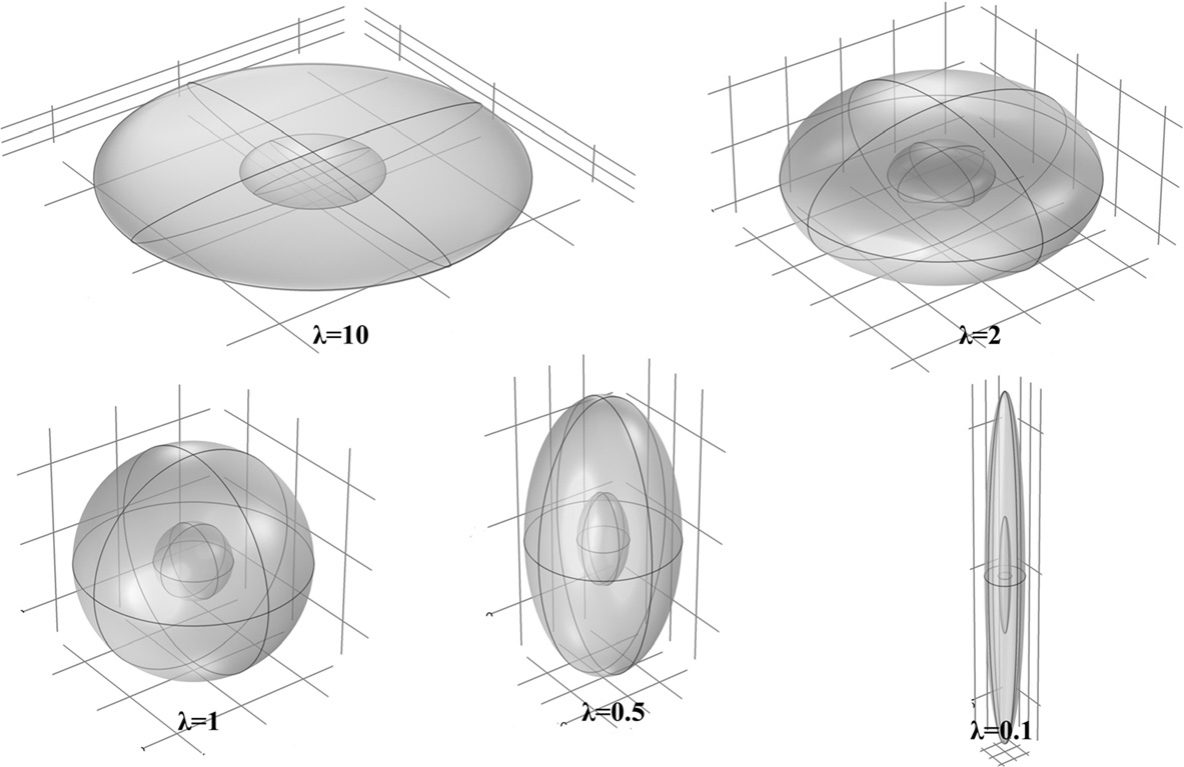
**Different shapes of solid tumors considered in this study.** The five ellipsoid shapes changed from oblate to prolate are studied.

To investigate the effect of tumor shape on drug delivery, 5 different shapes are considered. The 3D fundamental shape of tumors is assumed to be an ellipsoid in different studies. The assumption of considering ellipsoid tumor shape is investigated in many research such as breast cancer [47], prostate cancer [48,49] cervical cancer [50], glioma cancer [51], and others [52]. Based on this mentioned reason, ellipsoidal shapes of tumors are considered in this study. Different shapes are produced by changing ratio of two radii of ellipsoid shown in Figure 1. This ratio (*λ* = *b*/*a*) is changed from 0.1 (prolate) to 10 (oblate). In all shapes shown in Figure 16 the volume of the tumor is remained constant and equal to the volume of spherical tumor with radius *R*. The baseline value of *R* is 1 cm. The values of *R* used in sensitivity analysis are changed from 0.1 cm to 2.5 cm. This range is obtained from the literature and is close to the experimental observations [40].

### Model parameterization

The interstitial transport properties for normal and tumor tissue are listed in Table 1. These values are used as baseline and some of them are investigated and changed in specified ranges for sensitivity analysis.

The parameters of solute transport model taken from Baxter and Jain [8] are listed in Table 2. Although, the numerical model is applicable for any type of solute, in present study the properties of Fragment antigen-binding (F(ab’)_2_) as a sample is used.

### Numerical solution

**Figure 17:**
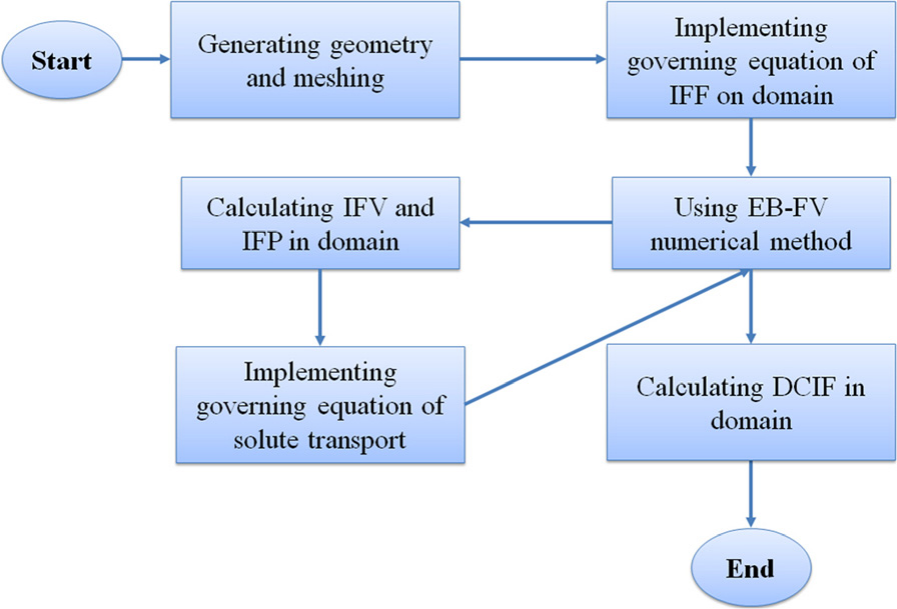
Algorithm of numerical simulation used for calculating interstitial fluid flow parameter (IFV, IFP) and solute transport parameter (DCIF).

A systematic flow chart is illustrated in Figure 17 to clarify the computational techniques involved in this paper. The criterion for the convergence of iterative solution based on element–based finite volume (EB-FV) method is to reduce the residual by 6 orders of magnitudes. The details of numerical method is mentioned in our previous works [2,16,53].

## Abbreviations

CFD: Computational fluid dynamics; IFP: Interstitial fluid pressure; IFV: Interstitial fluid velocity; DCIF: Drug concentration in interstitial fluid; EB-FV: Element based- finite volume.

## Competing interests

The authors declare that they have no competing interests.

## Authors’ contributions

MS and MS carried out the numerical simulations and drafted the manuscript. HB participated in the numerical simulations. MM and MB and KR helped to draft the manuscript and discussed the results. MS and MS conducted the numerical simulation, discussed the results and reviewed the manuscript. All authors read and approved the final manuscript.
